# Supplementary material for: Complexome profiling of the Chlamydomonas psb28 mutant reveals TEF5 as an early PSII assembly factor
Source: Plant Cell. 2025 Mar 17;37(6):koaf055. doi: 10.1093/plcell/koaf055 (PMC12164590; doi:10.1093/plcell/koaf055)
Supplement: koaf055_Supplementary_Data [file koaf055_supplementary_data.zip › psb28_interactive.zip!/plots/intensity/qualityPlots/peptides/_Cre06.g302700.t1.1.html]

### Cre06.g302700.t1.1

Trivial name:   
SeqCoverage [median]: 29.40  
Mass: 57.37 kDa  
  
 

| Sequence | MissedCleavage | Mass [Da] | wtIntensity | muIntensity | Score |
| --- | --- | --- | --- | --- | --- |
| ANPEGAPR | 0 | 810.40 | 1245.31 | 3553.05 | 75.11 |
| FFASVGSGAR | 0 | 997.50 | 0.00 | 844.63 | 70.92 |
| GLSVLDLGGNNIGPK | 0 | 1452.79 | 0.00 | 1649.06 | 91.55 |
| GNSLGNDGAILLSR | 0 | 1385.73 | 2801.26 | 9515.87 | 165.72 |
| GVAVLAGALR | 0 | 925.57 | 7186.09 | 20455.47 | 117.16 |
| LDLSGNAIDK | 0 | 1044.55 | 0.00 | 665.51 | 56.92 |
| LNSNYITR | 0 | 979.51 | 3690.77 | 5800.56 | 119.41 |
| MAQSIADNK | 0 | 976.46 | 621.98 | 0.00 | 84.21 |
| MAQSIADNKGLSVLDLGGNNIGPK | 1 | 2411.25 | 0.00 | 1104.17 | 52.36 |
| NHGALSSLELGYNPLGPEGTK | 0 | 2153.08 | 0.00 | 1507.84 | 73.67 |
| SAVETLPQAVSQGMQQK | 0 | 1800.90 | 903.46 | 524.20 | 96.82 |
| SQHAVDLSAK | 0 | 1054.54 | 3903.78 | 5022.66 | 160.67 |
| VTSSSSDSASLR | 0 | 1195.57 | 1767.68 | 632.89 | 102.06 |
|  |  |  | 22120.3 | 51275.9 |  |
